# Long-Term Post-COVID-19 Associated Oral Inflammatory Sequelae

**DOI:** 10.3389/fcimb.2022.831744

**Published:** 2022-03-02

**Authors:** Areej Alfaifi, Ahmed S. Sultan, Daniel Montelongo-Jauregui, Timothy F. Meiller, Mary Ann Jabra-Rizk

**Affiliations:** ^1^Department of Oncology and Diagnostic Sciences, School of Dentistry, University of Maryland Baltimore, MD, United States; ^2^Department of Restorative and Prosthetic Dental Sciences, College of Dentistry King Saud bin Abdulaziz University for Health Sciences, Riyadh, Saudi Arabia; ^3^Greenebaum Cancer Center, University of Maryland, Baltimore, MD, United States; ^4^Department of Microbiology and Immunology, School of Medicine, University of Maryland, Baltimore, MD, United States

**Keywords:** COVID - 19, inflammation, oral, salivary glands, antimicrobial peptide, opportunistic infection

## Abstract

The oral cavity remains an underappreciated site for SARS-CoV-2 infection despite the myriad oral conditions observed in COVID-19 patients. Recently, replicating SARS-CoV-2 was found inside salivary epithelial cells resulting in inflammation and atrophy of salivary glands. Saliva possesses healing properties crucial for maintaining the health of the oral mucosa. Specifically, salivary antimicrobial peptides, most notable, histatin-5 exclusively produced in salivary glands, plays a vital role in innate immunity against colonizing microbial species. The demonstration of SARS-CoV-2 destruction of gland tissue where histatin-5 is produced strongly indicate that histatin-5 production is compromised due to COVID-19. Here we present a case of a patient presenting with unexplained chronic oral dysesthesia and dysgeusia post-recovery from COVID-19. To explore potential physiological mechanisms behind the symptoms, we comparatively analyzed saliva samples from the patient and matched healthy subject for histatin-5 and key cytokines. Findings demonstrated significantly reduced histatin-5 levels in patient’s saliva and activation of the Th17 inflammatory pathway. As histatin-5 exhibits potent activity against the opportunistic oral pathogen *Candida albicans*, we evaluated saliva potency against *C. albicans ex vivo*. Compared to control, patient saliva exhibited significantly reduced anti-candidal efficacy. Although speculative, based on history and salivary analysis we hypothesize that salivary histatin-5 production may be compromised due to SARS-CoV-2 mediated salivary gland destruction. With the current lack of emphasis on implications of COVID-19 on oral health, this report may provide lacking mechanistic insights that may lead to reassessment of risks for oral opportunistic infections and mucosal inflammatory processes in acutely-ill and recovered COVID-19 patients.

## Introduction

The oral cavity remains an underappreciated site for SARS-CoV-2 infection despite the evident myriad oral manifestations in COVID-19 patients and the presence of SARS-CoV-2 in saliva (Gherlone et al., 2021; Huang et al., 2021). The oral conditions reported to be associated with COVID-19 include white and erythematous plaques, blisters, necrotizing gingivitis, ulcerations, salivary gland alterations, gustatory dysfunction and coinfections due to hypergrowth of opportunistic oral pathogens (Dos Santos et al., 2020; Salehi et al., 2020; Riad et al., 2020; Amorim Dos Santos et al., 2021; Brandini et al., 2021). Yet to date, little is known about the physiological mechanisms of oral manifestations in COVID-19 disease, and the impact of infection on salivary gland function.

The salivary glands were recently reported to be a potential target for SARS-CoV-2 infection due to the demonstrated expression of ACE2/transmembrane serine proteases 2 (TMPRSS2) receptor in salivary glands epithelial cells (Pascolo et al., 2020; Corchuelo and Ulloa, 2020; da Silva Pedrosa et al., 2021; Tsuchiya, 2021). Most notable however, are findings from a recent landmark study by Huang et al. (2021), demonstrating the presence of replicating SARS-CoV-2 in ducts, serous and mucous acini of salivary glands. Additionally, immunophenotyping and microscopic analysis demonstrated chronic and focal lymphocytic sialadenitis with predominance of T lymphocytic inflammation. Significantly, architectural distortion, atrophy, fibrosis, and ductal rupture were also observed thus establishing minor and major salivary glands as susceptible sites for infection, replication and local immune cell activation. In fact, clinical observations are in support of SARS-CoV-2 mediated damage to the salivary glands, as COVID-19 patients frequently present with gustatory dysfunction, and clinical cases of salivary gland changes have been reported (Gherlone et al., 2021). Collectively, these studies clearly establish the oral cavity as a robust site for SARS-CoV-2 infection with potential long-term effects on the health of the oral mucosa.

The homeostasis of the oral cavity is maintained by saliva, an extracellular fluid produced by salivary glands, and secreted in the mouth through openings called salivary ducts (Vila et al., 2019). Saliva possesses a wealth of protective and healing properties, particularly in defense against microbial inhabitants of the oral cavity, commensals and pathogens alike, as it is rich with diverse antimicrobial compounds (Salvatori et al., 2016; Vila et al., 2019). Specifically, host-produced salivary antimicrobial peptides play a vital role in innate immunity as they constitute the first line of defense against microbial species (Salvatori et al., 2016; Vila et al., 2019). The histatins in particular are a set of antimicrobial and anti-inflammatory peptides with wound healing properties considered to be crucial for maintaining the health of the oral mucosa (Peters et al., 2010; Sultan et al., 2018; Vila et al., 2019). Histatins are unique as they are exclusively produced and secreted into saliva by minor and major salivary glands of humans; specifically, histatins are localized in the serous acinar cells and intercalated duct cells, where they are stored in the secretory granules of the serous cells and are released by exocytosis at the luminal cell surface (Oppenheim et al., 1988; Ahmad et al., 2004). Histatin-5 (Hst-5) is the most abundant and notable member as it uniquely exhibits potent killing activities against the fungal pathogen *Candida albicans* (Edgerton et al., 2000; Helmerhorst et al., 2001; Ahmad et al., 2004). Although *C. albicans* is a commensal oral colonizer, if there is disruption in host environment, this species can rapidly transition into a pathogen causing oral candidiasis, the most common oral opportunistic infection, particularly in immunocompromised individuals (Jabra-Rizk et al., 2016; Vila et al., 2020)

The recent demonstration of SARS-CoV-2 replication in salivary glands and destruction of gland tissue integrity, including sites where histatins are produced and stored, strongly indicate that histatin production and secretion could be compromised in COVID-19 patients. Therefore, it is reasonable to speculate that COVID-19 patients with decreased Hst-5 salivary levels may be predisposed to development of candidiasis or oral inflammatory conditions. Yet despite the intense interest in COVID-19, the impact of the infection on host salivary innate immune factors and the mechanisms behind the reported oral manifestations, remain unknown. Here we present a case of long-term post-COVID-19 associated oral inflammatory sequelae in a patient presenting with unexplained chronic oral symptoms following recovery from COVID-19. Analysis of saliva samples recovered from the patient revealed significant reduction in Hst-5 levels and compromised salivary anti-candidal efficacy with indication for activation of the Th17 inflammatory pathway. With the current lack of emphasis on the implications of COVID-19 on oral health, this report may provide lacking mechanistic insights that may lead to reassessment of the risks for oral opportunistic infections and inflammatory processes in acutely ill and recovered COVID-19 patients.

## Materials And Methods

### Subject

A 48-year-old otherwise healthy female contracted COVID-19 in January 2021. The patient was a never-smoker and her past medical history was non-contributory. In March 2021, the patient developed “pins and needles” sensation of her fingertips and oral symptoms including taste alterations and oral dysesthesia (abnormal burning sensation). Of note, her tongue burning intensified and flared following administration of her first dose of the COVID-19 vaccine in April 2021. In June 2021, the patient presented to the Oral Medicine Clinic at the University of Maryland School of Dentistry with continued oral symptoms of oral dysesthesia and dysgeusia (altered taste sensation). The patient reported that her burning was confined to her tongue dorsum (especially the tip of her tongue) and her hard palatal mucosae. She described a constant low-grade burning sensation with the burning intensifying in the evening ranging from “mild” to “severe intense burns,” with occasional symptom-free days. Extra- and intra- oral examinations did not reveal any evidence of erythema or ulcerations. Her salivary flow was also within normal limits, and she had good floor of mouth salivary pooling and saliva was readily expressed for Stenson’s duct bilaterally. Mouthwash rinses containing lidocaine and diphenhydramine provided temporary relief in her oral symptoms for up to 4 hours. At a follow-up visit in September 2021, the patient reported 25% improvement in her oral symptoms but continued to experience persistent low-grade tongue dorsum burning although her altered taste returned to normal. Based on her clinical symptoms and history, she was diagnosed with post-COVID-19 associated oral dysesthesia, with her taste alterations likely secondary to inflammatory insult to the taste buds.

### Clinical Samples

At each of the two visits to the clinic (3 months apart), unstimulated whole saliva samples were collected for microbial culturing as well as a sample collected using the Salivette collection systems for salivary histatin-5 and cytokine measurement. A standardized method of 2-minute whole saliva sample collection was used to measure salivary flow rate (ml/min). Samples were also recovered from a healthy age and gender matched volunteer. Immediately following collection, whole saliva samples were cultured on fungal Yeast Peptone Dextrose (YPD) agar media (Difco Laboratories) and plates were incubated at 35°C for 24-48 hrs for assessment of *Candida* colonization status. Samples collected with the Salivette system were clarified by centrifugation, aliquoted and stored at -80°C with protease inhibitors for Hst-5 levels and cytokine analysis. Saliva samples were similarly collected from the control subject on two equally spaced separate occasions.

### Measurement of Hst-5 Salivary Levels Using ELISA

ELISA was performed as we previously described (Khan et al., 2013); Hst-5 peptide was synthesized by GenScript and Hst-5 specific polyclonal antibody was produced by Lampire Biological Laboratories. For measurement of Hst-5 levels, a standard curve was performed with each assay using Hst-5 peptide concentrations ranging from 0.5-500μg/ml. Wells of high-binding 96-well plates were coated with 100μl of each Hst-5 concentration or 1/100 dilution of saliva. Following overnight incubation at 4°C, wells were blocked with 0.1% dry milk in PBS for 1 hr incubation, and anti-Hst-5 antibody (1/1000) (100μl) was added for 1 hr at 37°C. Following washing, HRP-labeled goat anti-rabbit secondary antibody (1/3000) (Abcam) was added and plates incubated for 1 hr at 37°C. Following washing, 100μl of ABTS Peroxidase Substrate (KPL, Inc.) was added and plates incubated for 20 mins until color develops. The reaction was stopped by the addition of 50μl of Stop Solution (KPL, Inc.) and optical density (OD) was measured at 405nm using a microtiter plate reader. A standard curve was plotted with each run; samples were tested in triplicate on three separate occasions and the average Hst-5 concentration calculated in μg/ml. Measured Hst-5 levels were corrected for dilution factors.

### *Ex Vivo* Candidacidal Efficacy of Saliva Samples With Predetermined Hst-5 Concentration

The two patient saliva samples and control subjects with predetermined Hst-5 concentrations were tested for anti-candidal ability as we previously performed (Khan et al., 2013) using the standard *C. albicans* SC5314 strain (Gillum et al., 1984). Briefly, *C. albicans* cultures were grown in YPD broth (Difco Laboratories) overnight at 30°C and washed cells were resuspended in PBS. For these assays, saliva samples were filter-sterilized then added (100µl) to the wells of 96-well microtiter plates with *C. albicans* cells to final cell density of 1x10^4^ cells/ml. Cells in PBS or Hst-5 purified peptide (5 μg/ml and 20 μg/ml) (predetermined concentration from previous study based on significant killing activity) in PBS were included as negative and positive controls, respectively. Following 1hr incubation at 37°C with shaking, aliquots from reactions were diluted and plated on YPD agar and incubated for 24-48 hrs at 35°C. The number of colonies was counted and percent cell killing calculated based on drop in CFU counts compared to the control (PBS).

### Profiling of Saliva Pro- and Anti-Inflammatory Markers

As salivary cytokines provide valuable information on oral inflammatory conditions, multiplex cytokine analysis was performed on all saliva samples at the University of Maryland Cytokine Core using the Luminex Multianalyte System. Each sample was measured in triplicate and results expressed in Pg/ml.

## Results

### Salivary Histatin-5 Levels

All saliva samples cultured (patient and control subject) were negative for fungal culture indicating no commensal colonization by *Candida*. For Hst-5 levels, the average concentrations for patient samples #1 and #2 (collected 3 months apart) were 1µg/ml and 2.3 µg/ml, respectively. In contrast, Hst-5 concentrations for control subject samples #1 and #2 (collected 3 months apart) were 21.3 and 19.6 µg/ml, respectively (**Table 1**). On average, the patient’s Hst-5 values were approximately 92% lower than those of the healthy control subject.

**Table 1:** Comparative Hst-5 (µg/ml) and cytokine (Pg/ml) levels in saliva samples from patient and control subject.

Sample	IL-17A	IL-23	IL-6	IL-22	GMCSF	IL-18	TNF-a	IL-1B	Hst-5
**Control subject**	ND	ND	ND	ND	2.23	10.09	1.09	26.33	20.5
**Patient sample #1**	0.34	2.95	ND	4.82	0.31	1.45	0.05	1.02	1.0
**Patient sample #2**	0.93	10.08	0.74	21.49	0.80	2.42	0.02	2.68	2.3

ND, not detectable.

### Histatin-5 Concentration Dependent Anti-Candidal Salivary Activity

**Figure 1:**
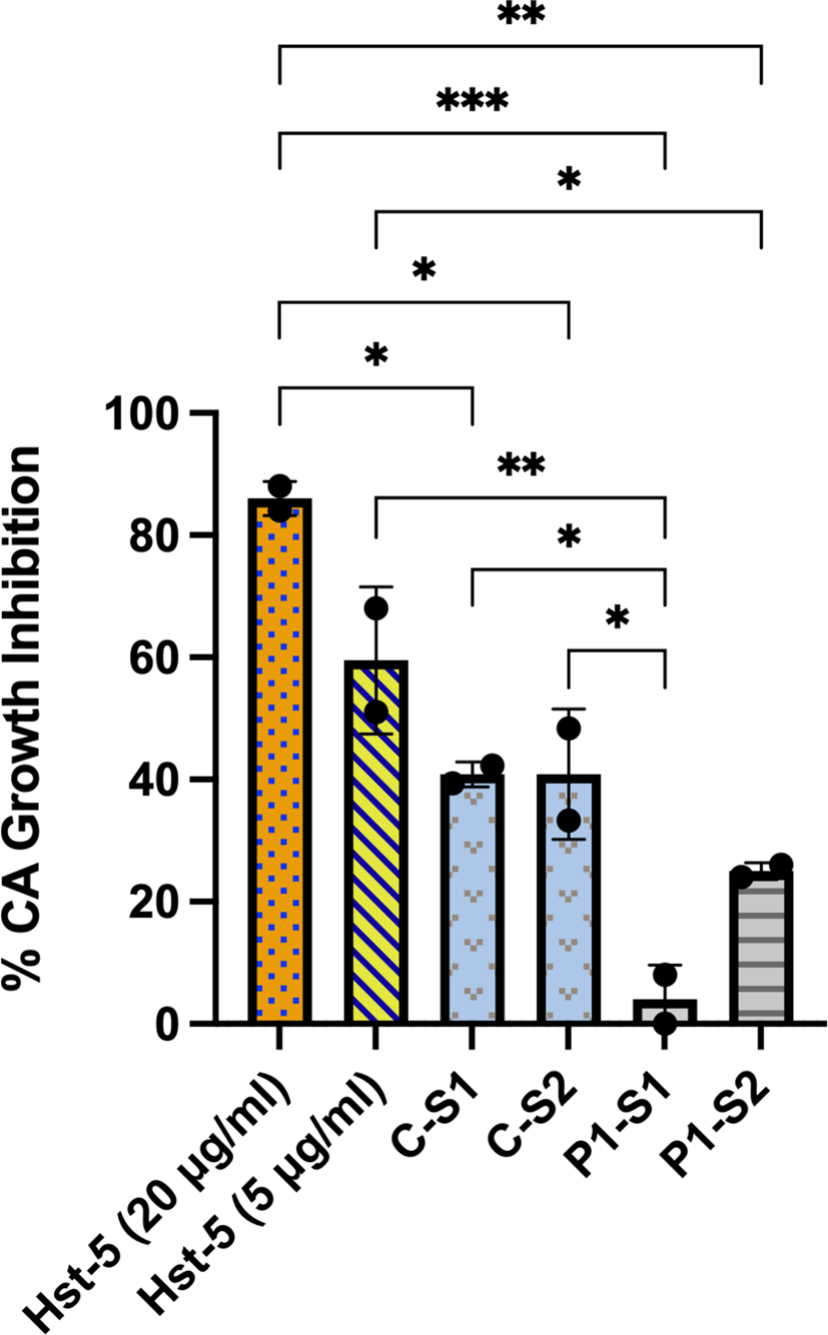
*Ex vivo* evaluation of anti-candidal activity of patient and control subject saliva based on reduction in *C. albicans* CFU counts. When the patient (P) and control subject (C) saliva samples with pre-determined Hst-5 levels were used in our *Candida* killing assay, both patient samples exhibited significantly reduced inhibitory effect on *C. albicans* compared to the control samples. The percent killing of the patient samples was proportional to measured Hst-5 concentration (P-S1: 1µg/l; P-S2: 2.3µg/ml). Purified Hst-5 peptide (5-20 µg/ml) was used as positive control for killing, and PBS as negative control. *P = 0.03; **P < 0.0021; ***P < 0.0002.

When the patient and control subject saliva samples with pre-determined Hst-5 levels were used in our *in vitro Candida* killing assay, both patient saliva samples exhibited significantly lower killing anti-candidal activity compared to the control sample based on reduction in *C. albicans* CFU counts following incubation in saliva; patient sample #1 with lowest Hst-5 concentration resulted in approximately 4% reduction in *C. albicans* growth and sample #2 resulted in approximately 25% reduction in *C. albicans* growth. In contrast, the saliva samples from the healthy control subject resulted in approximately 41% reduction in *C. albicans* growth, consistent with what has previously been established as within normal physiological Hst-5 concentration and anti-candidal saliva activity (Khan et al., 2013) (**Figure 1**).

### Cytokines Analysis

Comparative evaluation of salivary cytokine levels in the patient’s and control subject’s samples demonstrated an increase in the Th17 associated inflammatory cytokines (IL-17, IL-22, IL-23) in both patient’s samples. In contrast, GMCSF, IL-18, IL-1B and TNF-alpha levels were lower in the patient’s samples, whereas INF-gamma, IL-10, IL-17E, IL-17F levels were not detectable in all samples analyzed (**Table 1**).

## Discussion

The exact role of SARS-CoV-2 in the development of symptomatic oral conditions remains unclear. Innate immunity represents the first line of defense and provides the initial host response to tissue injury, trauma, and pathogens. Further, innate immunity activates the adaptive immunity, and both act highly regulated together to establish and maintain tissue homeostasis. Any dysregulation of this interaction can result in chronic inflammation and is thought to be a major underlying cause in the initiation and progression of immune-mediated oral inflammatory diseases such as periodontitis (Bunte and Beikler, 2019). Studies investigating salivary cytokines have demonstrated their utility as diagnostic biomarkers for oral inflammatory conditions such as Sjögren’s syndrome, periodontitis and oral infections (Diesch et al., 2021). Importantly, asymptomatic SARS-CoV-2 infected subjects were shown to exhibit consistent salivary IgG antibodies against SARS-CoV-2 indicating sustained, local immune responses in saliva (Huang et al., 2021). Therefore, in this study, we analyzed levels of Hst-5 and key cytokines in prospective samples recovered from a patient and a matched control subject. Comparative evaluation demonstrated significant reduction in Hst-5 levels in both of the patient’s samples, which was concomitant with activation of the Th17 inflammatory pathway (**Table 1**).

Interestingly, in a clinical study, we had previously demonstrated salivary Hst-5 levels to be significantly reduced in a cohort of HIV^+^ individuals compared to healthy control individuals, likely due to HIV related salivary dysfunction (Khan et al., 2013). Significantly, the decrease in salivary Hst-5 levels was concomitant with enhanced *Candida* colonization and compromised anti-candidal salivary activity proportional to Hst-5 concentration. In subsequent studies, in addition to demonstrating the importance of this salivary peptide in protection against *Candida* proliferation, we also demonstrated wound healing activity for Hst-5 on an oral cell line wound model (Kong et al., 2015; Sultan et al., 2019). Similar to the HIV study, here, the Hst-5 levels in both of the patient’s samples were significantly lower compared to the matched healthy control and importantly, the anti-candidal potency for the patient’s saliva was also in line with what we had previously seen relevant to Hst-5 concentration. *Candida* was not recovered from cultures of the patient saliva at the time of sampling; however, up to 70% of individuals are commensally orally colonized with *Candida* at any given time (Gerós-Mesquita et al., 2020). Nevertheless, our analysis indicates that present or future colonization with *Candida*, would put the patient at risk of developing oral candidiasis.

Th17 cells and the interleukin (IL)-17/IL-23 axis play pivotal roles in the pathogenesis of highly prevalent immune-mediated inflammatory diseases (IMIDs) such as periodontitis (Bunte and Beikler, 2019). In fact, IL-23 is considered a key cytokine for the pathogenesis of inflammatory and autoimmune diseases. The pathological consequences of excessive IL-23 signaling have been linked to its ability to promote the production of inflammatory mediators, such as IL-17, IL-22 by Th17 and IL-17-secreting cells (Pastor-Fernández G and Navarro, 2020). Similarly, IL-17 is a proinflammatory cytokine that is pathogenic in autoimmunity and inflammatory conditions, and abnormalities in IL-17 can promote the production of pro-inflammatory cytokines and aggravate autoimmune disorders (Zhang et al., 2018). Interestingly, IL-22, IL-23 and IL-17 were shown to be significantly increased at both protein and mRNA levels in inflamed salivary glands of patients with primary Sjögren’s syndrome (pSS), indicating that the Th17/IL-23 system may play a pro-inflammatory role in the pathogenesis of pSS (Ciccia et al., 2012). Furthermore, IL-22 and IL-23 levels were also shown to be significantly higher in patients with oral lichen planus, another chronic oral inflammatory disease (Nguyen et al., 2008; Chen et al., 2013; Mardani et al., 2021). Importantly, increasing evidence suggests a potential role for the IL-23/17 axis in the pathogenesis of COVID-19 *via* activation of cytokine cascade, and circulating IL-17 levels were found to be higher in COVID-19 patients, particularly critically ill COVID-19 levels (Martonik et al., 2021; Jahaj et al., 2021).

**Figure 2:**
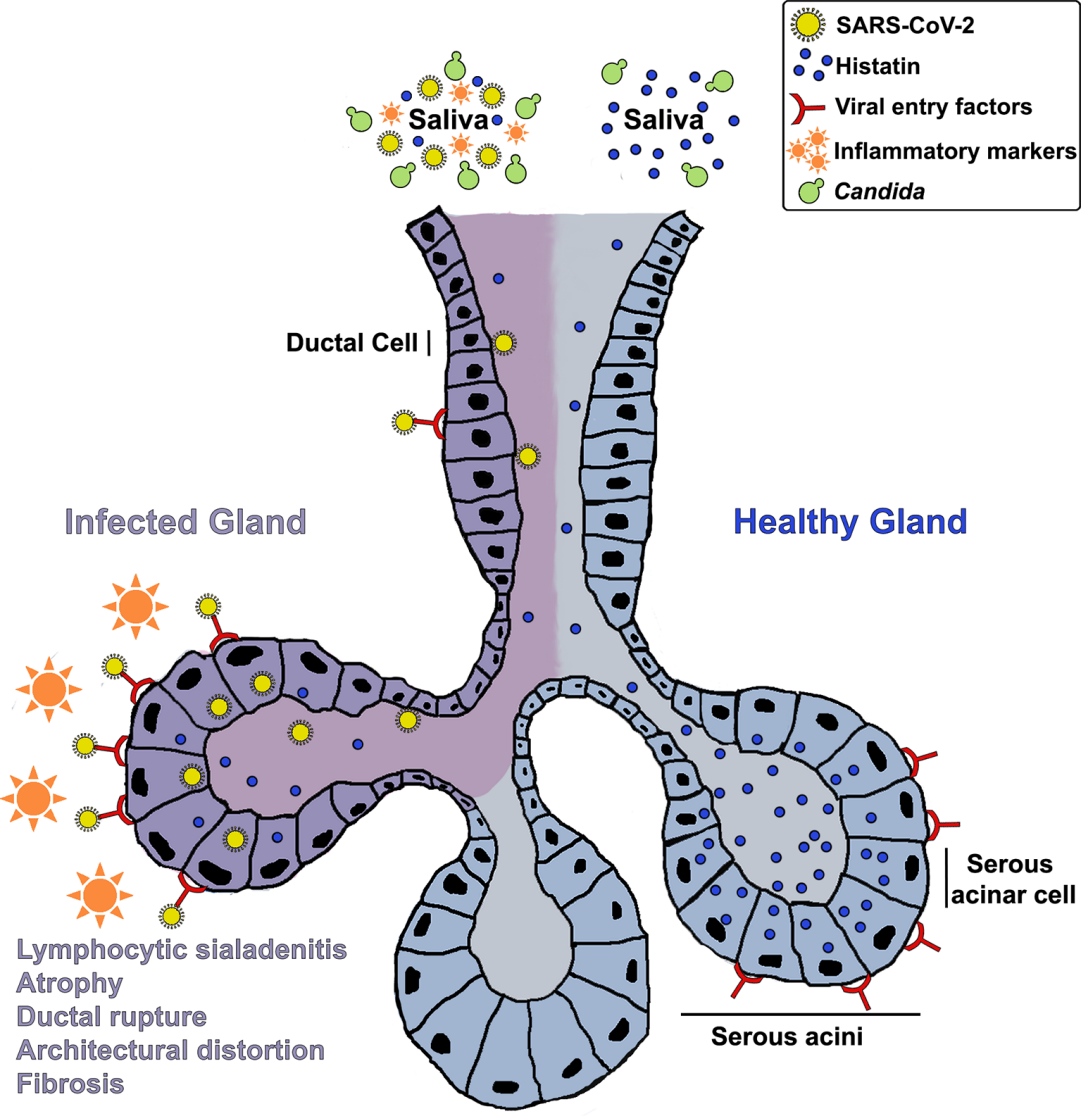
A hypothetical mechanistic illustration of the impact of COVID-19 on salivary glands and histatin production and secretion into saliva potentially predisposing patients to opportunistic infections and mucosal tissue inflammation. A schematic of a salivary gland depicting: Enrichment of viral entry factors (receptors) and replication of SARS-CoV-2 in gland serous acini and ducts; Resulting lymphocytic sialadenitis, architectural distortion, ductal rupture and atrophy of infected SG; Localization of histatin in the serous acinar and duct cells; Release of histatin and inflammatory cytokines into saliva.

Although speculative at this point, in light of the lack of any confounding factors that may explicate the onset of an oral inflammatory or neuropathic condition in an otherwise healthy individual, it is reasonable to posit that the oral symptoms experienced by the patient may have been mediated by lasting effects of an over-activated immune response due to SARS-CoV-2 infection. These speculations linking the oral condition to the virus are supported by the fact that the patient experienced a flare up of her symptoms following administration of the first dose of the COVID-19 vaccine. Overall, based on clinical history and salivary analysis, we hypothesize that histatin production and secretion into saliva may be compromised in COVID-19 patients due to SARS-CoV-2 mediated salivary gland destruction, potentially predisposing patients to long term opportunistic infections and mucosal inflammatory conditions, well after clinical recovery (**Figure 2**). However, further large-scale studies are needed using various COVID-19 patient cohorts to elucidate the connection between SARS-CoV-2 infection and oral disorders, and the effect of the inflammatory response on oral homeostasis.

## Data Availability Statement

The original contributions presented in the study are included in the article/supplementary material. Further inquiries can be directed to the corresponding author.

## Ethics Statement

The studies involving human participants were reviewed and approved by University of Maryland Baltimore Institutional Review Board. Written informed consent for participation was not required for this study in accordance with the national legislation and the institutional requirements.

## Author Contributions

Conceptualization: MJ-R, AS, and TM. Methodology and investigation: AA and DM-J. Formal analysis: MJ-R, AS, AA, and DM-J. Writing (original draft): MJ-R and AS. Writing (review and editing): all authors. All authors contributed to the article and approved the submitted version.

## Funding

This work was supported by the National Institute for Health (NIDCR) under award number R21DE031888 MJ-R.

## Conflict of Interest

The authors declare that the research was conducted in the absence of any commercial or financial relationships that could be construed as a potential conflict of interest.

## Publisher’s Note

All claims expressed in this article are solely those of the authors and do not necessarily represent those of their affiliated organizations, or those of the publisher, the editors and the reviewers. Any product that may be evaluated in this article, or claim that may be made by its manufacturer, is not guaranteed or endorsed by the publisher.
